# Preparation, biocontrol activity and growth promotion of biofertilizer containing *Streptomyces aureoverticillatus HN6*

**DOI:** 10.3389/fpls.2022.1090689

**Published:** 2022-12-15

**Authors:** Tianhao Wang, Shakil Ahmad, Lin Yang, Xiangnan Yan, Yunfei Zhang, Shujing Zhang, Lanying Wang, Yanping Luo

**Affiliations:** School of Plant Protection, Hainan University, Haikou, Hainan, China

**Keywords:** *Streptomyces aureoverticillatus HN6*, biofertilizer, biocontrol, frame composting, natural composting, plant growth

## Abstract

Nowadays, due to the excessive dependence on chemical fertilizers and pesticides in agricultural production, many problems, such as soil hardening and soil-borne diseases, have become increasingly prominent, which seriously restrict the sustainable development of agriculture. The application of microbial fertilizer prepared by biocontrol microorganisms can not only improve soil structure and increase fertility but also have the function of controlling diseases. *Streptomyces aureoverticillatus HN6* has obvious disease prevention and growth promotive effect, which can improve the rhizosphere fertility of plants and even regulate the rhizosphere microbial community of plants. Based on the comparison of frame composting and natural composting, we used the response surface method to optimize the preparation conditions of *Streptomyces HN6* bacterial fertilizer. The results showed that natural composting not only produced higher composting temperatures and maintained long high temperature periods in accordance with local conditions, but was also more suitable for composting in the field according to local conditions. Therefore, the substrate’s conductivity changed more, the ash accumulation increased, and the substrate decomposed more thoroughly. Thus, this composting method is highly recommended. Additionally, *Streptomyces HN6* microbial fertilizer EC20 can reduce cowpea fusarium wilt and promote cowpea growth. The number of plant leaves, plant height and fresh weight, increased significantly in the microbial fertilizer EC20. Moreover, *Streptomyces HN6* fertilizer EC20 could significantly induce soil invertase, urease and catalase activities. Our study highlights the potential use of *Streptomyces HN6* as a biofertilizer to improve plant productivity and biological control of plant pathogenic fungi.

## 1 Introduction

The exponential growth in the world’s population makes sustainable, safe, and secure food a priority with a minimum impact on the economy, earth, and the environment. A conventional farming system that relies heavily on inputs, however, leaves long-term damage to the production system, food quality, biodiversity, greenhouse gas emission, and health of the human population. Annually, vast amounts of crop residues are disposed of as husks and bran during refining processes or burned in the field after harvest (Abbas, 2012). China produces more than 700 million tons of agricultural residues annually from various crops, including rice, wheat, maize, and soybeans, which account for 20-30% of the world’s production (Xu et al., 2019). A suitable development technology has not yet been developed to enable crop straw to be disposed of other than by landfilling or burning. To limit the amount of residues, the natural form of crop production must be considered. An example of this type of crop production is organic farming, which involves using botanical fertilizers, green manure, biological fertilizers, and biopesticides to fertilize and manage crops in an ecologically safe way (Durán-Lara et al., 2020).

Plant growth and yield are significantly improved through composted organic fertilizers developed from farm waste (Whitbread et al., 2003; Sharma et al., 2021). Additionally, the use of microorganisms in compost will make it more effective and can be used in a large field. Thus, essential nutrients (bioactive compounds) or indirectly (microbes) can be added to the compost to improve its quality and effectiveness. Bacteria and organic matter are combined in bioorganic fertilizer (BOF), which offers better performance than organic matter or microorganisms alone (Li et al., 2021). The presence of bioactive compounds and microbes stimulates various biological processes in the plant and positively impacts its growth (Singh, 2014). Microbes-containing compost is biologically active and effective for seed germination, plant growth, soil rehabilitation, and disease control (Uzoh and Babalola, 2018; Kaur et al., 2019; Zahra et al., 2021). Composts can also be supplemented with exogenous microorganisms to produce mycorrhizal fertilizers, which improve soil structure, increase organic matter accumulation, and maintain indigenous microorganisms (Youssef et al., 2021; Mahapatra et al., 2022).

The Cowpea (*Vigna unguiculata* (Linn.) Walp.) is a legume crop widely cultivated worldwide that belongs to the family of dicotyledonous herbs, the legume family. Bacterial blight of Cowpea in China caused by *Fusarium oxysporum* f.sp. tracheiphilium has been reported to infest seedlings to adult plants during the growing period. To control cowpea blight, resistant plant varieties were used, crop rotations were rationalized, seeds were disinfected, better cultivation management was employed, and chemicals were used (Sreeja, 2014; Berger et al., 2016). However, pesticide-based disease control strategies are associated with several disadvantages, including resistance development, resurgence of pathogens, and residual toxicity (Ullah et al., 2022). As biocontrol microbes become more prevalent, microbial control is becoming viable, such as using metabolites or fertilizers produced by bacteria to combat cowpea blight. Several researchers have reported successful control of cowpea blight using fungi and bacteria, including Campanella and Miceli (2021), Keshavarz et al. (2019), and Kannan et al. (2019).

A microbial community amplicon sequencing analysis revealed that *Streptomyces HN6* was effective in restoring soil prokaryotic microbial diversity and controlling Fusarium populations, and the soil microbial community clustered into one group at the genus level with the healthy soil microbial community (Yang et al., 2021). Therefore, this strain has good value for *in vivo* application. Using the response surface method, we optimized the conditions for *Streptomyces HN6* to grow in the substrate for subsequent fertilizer preparation by comparing the composting efficiency of box composting and natural composting. The microbial fertilizer was further evaluated for its efficacy against cowpea wilt disease and plant growth promotion effect in a pot experiment.

## 2 Materials and methods

### 2.1 Composting methods

#### 2.1.1 Fermentation solution preparation

A 2L PDB medium was inoculated with thermophilic siderophores and white rot and incubated at 120 rpm, 25°C for 7 days (Wang et al., 2012). The strain of *Streptomyces HN6* (CCTCC No. M2010293) obtained from the School of Plant Protection of Hainan University was inoculated in a 5L shaker flask at 120 rpm, 28°C for 7 days.

(i) Frame composting
In a homemade rectangular plastic frame, 80 kg of dried and crushed plant straw were inoculated at 10% with 1 x 10^9^ cfu mL^-1^ *S. thermophile* and *Phanerochaete chrysosporium* fermentation solution, then covered with black plastic film and composted (Chen et al., 2020). The pile temperatures were monitored twice daily, at 9:00 a.m. and 3:00 p.m. According to the temperature of the pile, the composting stage was determined, with the pile being heated, maintained at a high temperature (55°C for at least 3 days), then cooled as the maturation stage, and then aged at a constant temperature between 25°C and 40°C. The pile was aged at 10% inoculation with a suspension of *Streptomyces* sp. *HN6* spores for 21 d. The final microbial fertilizer was labeled EC19.
(ii) Natural composting method
In the natural composting process, 10% inoculum was added to crushed straw along with 1×10^9^ cfu mL^-1^ inoculum of thermophilic siderophore fermentation solution and 1×10^9^ cfu mL^-1^ white rot fermentation solution, mixed uniformly, and composted into a cone measuring 100 cm diameter and 30 cm height, repeated three times (Zhang et al., 2016). Temperature monitoring and turning times were the same as for frame composting. The fermented substrate was then mixed and divided into 3 portions, one inoculated with 1×10^9^ cfu mL^-1^ *Streptomyces HN6* at 10% inoculum and recorded as Bacterial fertilizer BZ20. Because of aging, one sample was naturally aged without microorganisms and recorded as Bacterial Fertilizer DF20; the other sample was used for the *Streptomyces HN6* response surface optimization test. The physical and chemical properties of the substrate, the enzyme activity, and the degree of decay during composting were tested in the same way as for frame composting.

#### 2.1.2 Determination of physicochemical parameters of the fertilizer

Compost minerals can be determined indirectly by electrical conductivity, pH changes, and nitrogen sources *via* changes in electrical conductivity and pH (Atkinson et al., 2010; Abdel-Fattah, 2012). Thus, we measured the EC and pH of both composts. The sample’s pH and electrical conductivity (EC) were determined by incubating 2 g of the sample with 20 mL of distilled water at 25°C at 120 rpm for 2 hours (Pastuszczak et al., 2022). To determine pH and EC, the obtained supernatant was centrifuged for 10 minutes at 8000 rpm after incubation. The total organic carbon and total nitrogen of compost samples from different periods were measured using an elemental analyzer; 2000.0 mg of compost samples were added to a beaker and mixed with 5 mL of HCL solution at pH 1.0, 2.0, 3.0, 4.0, and 5.0, and dried at 105°C using an oven. To determine the total organic carbon and total nitrogen content of the sample, 25 mg of the sample was ground thoroughly and passed through an 80 mesh sieve. Ash was measured by sintering. The crucible was washed and boiled in 25% hydrochloric acid for 5-10 min, then placed in a muffle furnace for one hour at 550°C. When the furnace temperature dropped below 200°C, the crucible was transferred into a desiccator with crucible tongs, cooled to room temperature, weighed, and 5 g of dried sample was added and weighed again. The materials were calcined in a muffle furnace at 550°C for 4 hours until no carbon particles remained, then transferred to the desiccator at 200°C and cooled to room temperature. The searing process was repeated until the difference between the two weighing did not exceed 0.1 mg.

#### 2.1.3 Determination of enzyme activity in the substrate

Cellulase, urease, and fluorescein diacetate hydrolase were determined by UV spectrophotometric method as described previously (Ashfaq et al., 2017).

#### 2.1.4 Determination of base material

(i) Determination of infrared spectra of compost leachate
The compost extract’s infrared absorption spectrum can analyze the compost material structure changes, and can quickly evaluate the compost maturity (Carballo et al., 2008). In this study, the infrared absorption spectra of the extracts of frame compost and natural compost materials were monitored. The specific method is as follows: The above-obtained supernatant (section 2.12) was filtered through a 0.45 µm microporous filter membrane. The filtrate was lyophilized using a FreeZone plus freeze-dryer (Kansas, MO, USA), a small amount of the lyophilized sample was mixed and ground with KBr to make pressed tablets. The infrared spectra were recorded at wavenumbers ranging from 400 to 4000 cm-1 by a Thermo Nicolet Avatar-330 spectrometer (Madison, USA).
(ii) Determination of seed germination index (GI) of compost leachate
Seed germination index is a sensitive biological indicator for evaluating the toxicity of compost, and it can even be used as a sign of compost maturity (Yang et al., 2021). It is directly related to whether or not compost applied to the field will harm crops based on the seed germination index. Therefore, to evaluate this, a 250 mL conical flask, and 50 mL distilled water was mixed with 5 g of compost leachate and incubated on a shaker for 2 hours at 120 rpm. We placed 20 cowpea seeds in a 9 cm petri dish covered with filter paper, then added 10 mL of compost leachate. Three replicates of each treatment were performed with 10 mL of sterilized tap water as a control. Samples were incubated in the dark at 28°C in an incubator for 4 days. Germination rates were calculated using the following formula:
GI (%) = (treatment seed germination rate × treatment average root length) ÷ (control seed germination rate × control average root length) × 100 (Mahmoodzadeh et al., 2015).

### 2.2 Test of *Streptomyces HN6* response surface

The Box-Behnken response surface design method was used to optimize the composting conditions of the strains based on the C/N, pH, and water content factors that influence fermentation conditions in the *Streptomyces HN6* response surface test (Wei et al., 2020). **Table S1** illustrates the levels of variation of the factors, where earthworm manure was used to adjust the C/N of the aging substrate, ferrous sulfate and calcium carbonate solutions were used to adjust the pH of the aging substrate, and distilled water was added to adjust the water content. The fermentation time of the compost was 21 d. The amount of *Streptomyces HN6* per gram of compost was measured using the gradient dilution method as an indicator of the fermentation effect of the compost. Lastly, natural compost material was used as an aging substrate, with the C/N, pH and water contents adjusted according to the optimal combination predicted by the response surface model, and *Streptomyces HN6* was added for fermentation verification and aging verification to get compost EC20.

Determination of Streptomyces HN6 content in the obtained bacterial fertilizer: To prepare the sample, 2 g were added to 20 mL of distilled water, then shaken at 25°C for 2 h at 120 rpm, followed by centrifugation at 8000 rpm min^-1^ for 10 min. In that order, 1 mL of supernatant was diluted to 10^-7^, 10^-8^, and 10^-9^. Aliquots of 25 μL were spread onto Koch’s One solid medium using a bent rod (Wang et al., 2021b). The culture was spread evenly and incubated for 5 d at 28°C. The number of bacteria contained in the unit sample was counted.

### 2.3 Application of biological bacterial fertilizer

#### 2.3.1 Growth-promoting effect of biological bacterial fertilizer on cowpea

Seeds of cowpea were treated with a warm blanching method, then sown in seedling trays and incubated until two leaves appeared. In plastic pots with a diameter of 17 cm, cowpea was given EC19 bio-fertilizer obtained from frame composting and BZ20, DF20, and EC20 obtained from natural composting. Cowpea seedlings were transplanted using the root dipping method inoculated with cowpea blight, one cowpea seedling per pot, and 15 plants per treatment. The treatment without bio-fertilizer and without cowpea wilt inoculation was used as control 1 (denoted as CK1), and the treatment without bio-fertilizer but with cowpea wilt inoculation was used as control 2 (denoted as CK2). Treatments were incubated in a greenhouse with natural light and regular irrigation. No additional fertilization was applied during the incubation period.

(i) Morphological indicators
In order to determine the number of leaf blades, plant height, stem thickness, root length, fresh weight, and dry weight of cowpeas 30 days after transplantation following (Nogueira et al., 2014; Asagba et al., 2017).
(ii) Physiological and biochemical indicators
Chlorophyll content, root vigor, soluble reducing sugar content, soluble protein content, catalase activity, and free amino acid content of cowpea seedlings were determined using the plant chlorophyll content test kit from Beijing Solabao Technology Co Ltd. Previously reported method were followed for TTC (Zhang et al., 2015), anthrone colorimetric, UV absorption and potassium permanganate titration (Wang et al., 2021b), ninhydrin color development (Lu and Mallette., 1970).
(iii) Several key enzymes in the soil
An analysis of soil biofertility was conducted by evaluating three soil enzymes, namely convertase, urease, and catalase. Specifically, convertase mediates the carbon cycle by catalyzing the hydrolysis of sucrose. Urease regulates the nitrogen cycle by catalyzing urea hydrolysis; and catalyzing the rapid breakdown of hydrogen peroxide to water and oxygen, which is known to be explained by the microbial activity in the soil (Wang et al., 2021a). The activities of these three enzymes at the sampling stage were assessed following (Sahu et al., 2010; Yan et al., 2021).

#### 2.3.2 Determination of bio-fertilizer efficacy against cowpea blight

After 30 days post-transplantation of cowpea seedlings, diseases incidence and diseases index of cowpea blight were calculated by using the formula incidence (%) = number of affected plants ÷ total number of plants × 100 and disease index = [Σ (number of affected plants × number of disease stages) ÷ (total number of plants × highest disease stage)] × 100. The biofertilizer’s efficacy was estimated using the formula efficacy (%) = (disease index of sterile water treatment - disease index of other treatments) ÷ disease index of sterile water treatment × 100%. The disease was graded according to (Wang et al., 2019), with the following criteria: Grade 0, no disease in the whole plant, no external symptoms; Grade 1, vascular bundles within 25% of the base turn brown; Grade 2, vascular bundles within the stem turn brown from 26% to 50%; Grade 3, vascular bundles within the stem turn brown from 51% to 75%; Grade 4, vascular bundles within the stem turn brown from 75% or more, or the whole plant dies of the disease.

### 2.4 Statistical analysis

Differences between treatments were calculated and analyzed using analysis of variance (ANOVA), Duncan’s multiple polar difference test, and t-test (P<0.05 or P<0.01). SPSS was used for all statistical analyses. In secondary fermentation condition exploration experiments, response surface experiments were designed and analyzed by Design-Expert 8.0. Pearson correlation coefficients were used to assess cultured medium cooking properties (temperature, pH, carbon to nitrogen ratio) and soil enzyme activity (including FDA enzymes, urease, and catalase).

## 3 Results

### 3.1 Temperature variation of different composting

**Figure 1:**
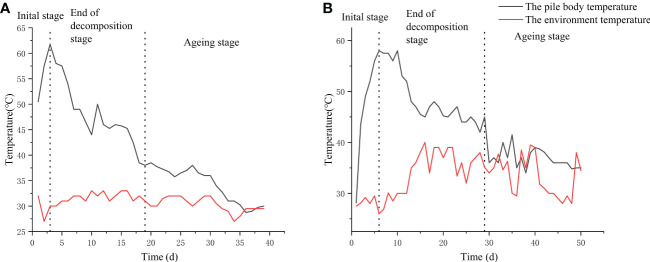
Changes in the temperature curve between ends of decomposition: **(A)** Frame composting; **(B)** Natural composting.

In this study, the changes in temperature of the base material between the frame composting method and the natural composting method were measured (**Figure 1**). The warming period of the frame composting was 3 d, and the high-temperature period was 4 d (at the 4^th^ day of composting, the highest temperature of the pile reached 61.8°C, and after turning and aerating the pile, the temperature of the pile gradually decreased to about 57°C for 3 d). The cooling period was 11 d (the high-temperature period gradually decreased to 40°C on the 18^th^ day; the temperature of the pile falls to ambient temperature after the 19^th^ day). After the aging phase, the pile was fed with a suspension of *Streptomyces HN6* spores and continued to ferment for 21 d, resulting in a frame compost EC19 (**Figure 1A**).

The natural composting frame compost had a warming period of 4 d and a high-temperature period of 8 d (the maximum temperature of the pile reached 58.8°C on the 5^th^ day of fermentation and was maintained at 55°C for 7 d after turning and oxygenating the pile). The cooling period was 17 d (after the high-temperature period, the temperature gradually dropped to below 40°C at day 29), and the aging stage was entered at 30 d (the pile temperature dropped to ambient temperature after day 30). The pile was divided into three parts after the aging phase, one part was added to the *Streptomyces HN6* spore suspension, and one part was aged naturally; both continued to age and ferment for 21 d to obtain fertilizers BZ20 and DF20, respectively (**Figure 1B**). The remaining part was used for *Streptomyces HN6* response surface optimization.

### 3.2 Effect of physicochemical parameters of different composting

The starting pH of frame composting was 7.07, and it rose to 9.08 at the end of the decaying stage and slowly decreased to 7.66 after the composting entered the aging stage. Natural composting started with a pH of 6.93, increased to 9.23 at the decaying stage, and decreased to 7.10 at the aging stage (**Table 1**). A 70% increase in conductivity was observed between the content of frame compost prior to decaying (877.0 μs·cm^-1^) and the content of frame compost after decaying (1473.5 μs·cm^-1^). The conductivity increased by 32.4% after the material was composted into the aging stage (1950.2 μs·cm^-1^). The conductivity of natural composting generally showed a continuous increase, rising by 78.7% at the end of the decomposition stage, and the conductivity rose from a starting level of 1044.4 μs·cm^-1^ to 2392.7 μs·cm^-1^, an increase of 129% by the end of composting.

**Table 1:** The result of physico-chemical parameters.

Composting method		pH	Electrical conductivity(μs·cm^-1^)	C/N	Ash(%)
Frame composting	Initial stage	7.07 ± 0.32b	877.0 ± 76.5c	25.22 ± 1.26a	4.33 ± 0.60c
	End of decomposition stage	9.08 ± 0.28a	1473.5 ± 75.6b	10.17 ± 0.65b	15.95 ± 1.21b
	Ageing stage	7.66 ± 0.19b	1950.2 ± 70.9a	9.17 ± 0.85b	18.21 ± 1.65a
Natural composting	Initial stage	6.93 ± 0.14b	1044.4 ± 14.2c	40.30 ± 7.22a	16.94 ± 1.45b
	End of decomposition stage	9.23 ± 0.37a	1866.7 ± 79.3b	25.17 ± 3.32b	20.88 ± 0.39a
	Ageing stage	7.10 ± 0.12a	2392.7 ± 143.5a	23.95 ± 5.17b	20.93 ± 0.34a

Data represent the mean ± SD of at least three independent experiments and different lower case letters indicate that differences between treatment groups reached significance at the P<0.05 level.

Before and after composting, frame compost material rapidly decreased from 25.22 to 10.17, indicating that the microbial metabolism was vigorous during fermentation, resulting in high carbon loss and good compost decay. During the aging stage, the carbon-to-nitrogen ratio decreased slowly, indicating that large amounts of organic matter were still decomposing in the pile, and fermentation and decay continued. *Streptomyces HN6* may have been involved in this process. By the end of the aging stage, the carbon to nitrogen ratio in the natural compost pile decreased by 37.5%, and by the end of composting, the carbon-to-nitrogen ratio decreased by 40.5% from its starting rate of 40.30% to 23.95%. Prior to and after the aging stage, the frame compost material’s ash content increased from 4.33% to 15.95%, with a significant increase indicating that the process was effective. As ash content did not change much during the aging stage, microorganisms were less likely to carry out mineralization during this period. The ash content of the natural compost increased by 23.2% at the end of the decomposition stage but did not change much after the aging stage.

### 3.3 Effect of enzyme activity in the substrate

**Figure 2:**
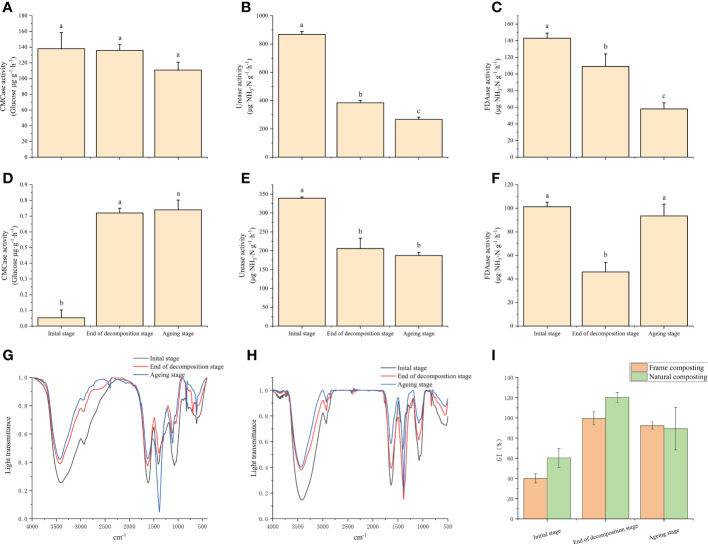
Changes in physicochemical parameters and decay indicators under natural and frame composting methods: **(A)** cellulose activity variation during frame composting; **(B)** urease activity variation during frame composting; **(C)** Changes in fluorescein diacetate hydrolase activity during frame composting; **(D)** cellulose activity variation during natural composting; **(E)** urease activity variation during natural composting; **(F)** Changes in fluorescein diacetate hydrolase activity during natural composting; **(G)** Infrared absorption spectra of material leachate at different stages of frame composting; **(H)** Infrared absorption spectra of material leachate at different stages of natural composting; **(I)** Effect of leachate on wheat seed germination index at different stages of frame composting and natural composting. Data represent the mean ± SD of at least three independent experiments, and different lowercase letters indicate that differences between different treatment groups reached significance at the P<0.05 level.

Various enzymes secreted by microorganisms play an important role in the degradation of macromolecules in compost substrates. In this study, the results indicate that the CMCase enzyme activity in the frame compost substrate was unchanged before and after the decaying stage and decreased significantly when entering the aging stage (**Figure 2A**). During the aging stage, the urease enzyme continued to decrease and decreased by 30.7% by the end of composting (**Figure 2B**). It was significantly lower at the end of the decaying stage compared to the beginning. A 23.8% decrease in FDAase was observed around the decaying stage of the enzyme, and this decrease continued into the aging stage (**Figure 2C**). Natural composting continued to increase CMCase enzyme activity during the ripening stage. However, the enzyme activity was not significantly different after the aging stage (**Figure 2D**). At the end of the ripening stage, urease enzyme activity decreased significantly from the beginning of the composting period and decreased by 39.4% (**Figure 2E**). The enzyme activity of FDAase was significantly lower at the end of the decaying stage than at the beginning of the composting period and then increased significantly at the end of the aging stage (**Figure 2F**). This change may be related to the addition of *Streptomyces* sp. *HN6* at the aging stage.

### 3.4 Effect of the degree of maturity of the base material

#### 3.4.1 The infrared spectrum of the composting leachate

We measured the infrared absorption spectra of frame compost and natural compost materials in this study (**Figures 2G, H**). There is no difference between the characteristic absorption peaks at the beginning of the composting period, the end of the decaying stage, and the end of the composting period, which appear at 3420, 2933, 1630, 1380, 1100, and 620 cm^-1^, respectively. As a result, the intensity of characteristic absorption peaks reflects the functional group level in the samples. The absorption peak at 3420 cm^-1^ is caused by the broad peak stretching vibration of -OH sourced from sugars. The intensity of the absorption peak here gradually decreases from 0 d to 39 d, especially before and after the decay stage, indicating that large molecules such as cellulose gradually decompose and are used mainly during the decay stage. The absorption peak at 2933 cm^-1^ is caused by symmetric or antisymmetric stretching vibrations of -CH_3_ and -CH_2_, originating from sugars, aliphatic compounds, and lignin, and the decrease in absorption intensity here also indicates the decomposition of cellulose, lignin, and other macromolecules during fermentation. The absorption peak at 620 cm^-1^ is caused by aromatic acids and decreases at the end of fermentation. Among the peaks, the 1380 cm^-1^ absorption peak is of particular interest and could be considered a marker for the complete decomposition of the material (Martinez-Sabater et al., 2009). The peak at 1380 cm^-1^ significantly strengthened at the end of the composting process, suggesting that the material in the frame compost pile does not mature completely until the aging stage. When compared to frame composting, natural composting shows a peak at 1380 cm^-1^, representing the compost’s full decomposition, which strengthens significantly at the end of the decomposition process. As a result, during the decomposition process, the base materials are more completely decomposed.

#### 3.4.2 Seed germination index of compost leachate

During the decaying stage of the frame compost, wheat seeds treated with base material leachate showed a significant increase in germination index from 40.1% to 99.5%, an increase of 148.1%, indicating that the base material was less toxic to plants during the decay stage (**Figure 2I**). The germination index of the wheat seeds treated with the base extract showed a trend of decreasing and then increasing, indicating that some toxic substances were produced by the heap during the aging process. Our results indicate that the final germination rate of wheat seeds treated with the composting process was 92.4%. A significant increase of 199.2% was observed for wheat seeds cultivated after natural composting, with a leachate-containing base material significantly increasing the germination index from 60.3% to 120.3%, without toxic effects.

### 3.5 Model stabilization

With A (water content), B (pH), and C (carbon to nitrogen ratio) as variables and *Streptomyces* secondary fermentation HN6 content as the response value, three parallel tests were extracted to take the average value (**Table S2**) and the results were obtained according to the combination of Box-Behnken Design experimental design provided by Response Surface Soft Design-Expert 8.0 (**Table S3**).

There is a good correlation between the regression equation and theoretical predictions of *Streptomyces* sp. *HN6* bacterial content in secondary fermentation. The values of A (water content), B (pH), and C (carbon to nitrogen ratio) under the optimal combination were obtained as 55.45%, 7.21 and 25.45 respectively, and the predicted effective bacterial content was 1.37 x 10^10^ cfu·g^-1^ by the computational response surface software.

### 3.6 Response surface and contours analysis

**Figure 3:**
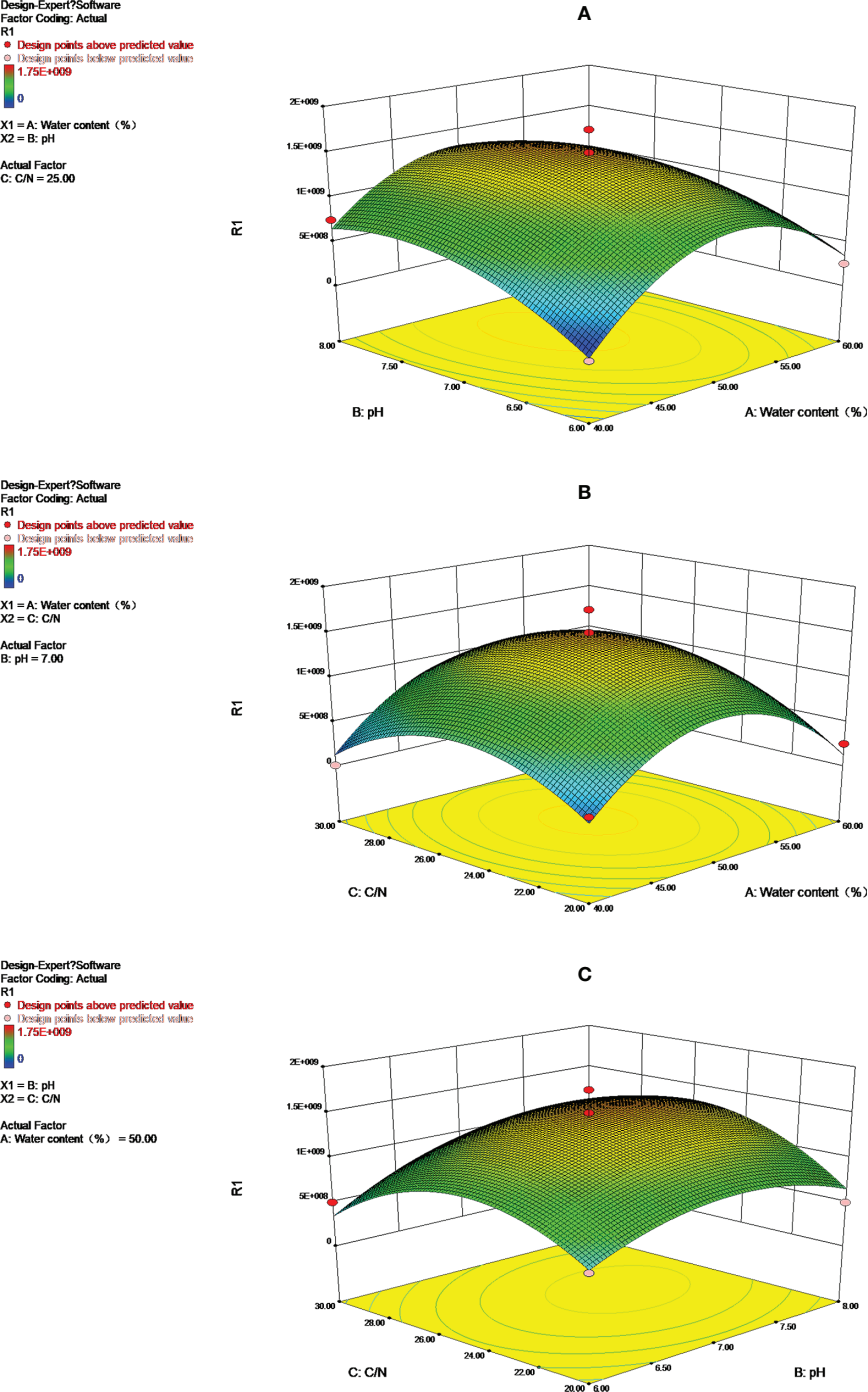
Response surface and contours to: **(A)** Y=f **(A, B)**; **(B)** Y=f **(A, C)**; **(C)** Y=f **(B, C)**.

An analysis of the effect of pH, carbon to nitrogen ratio, and water content on *Streptomyces HN6* aging fermentation was carried out by using the response surface and contour shape (**Figure 3**). Depending on the degree of urbanity of the surface interacting with the contour line, there will be greater interaction between the two factors. An ellipse indicates the presence of a significant interaction between the two factors. In a regression model, ANOVA with the response surface and contour plot, the bacterial content of *Streptomyces HN6* aging fermentation was greatly influenced by pH. However, there was no significant interaction between pH and water content, water content, and carbon to nitrogen ratios, but there was a strong interaction between pH and carbon to nitrogen ratios.

### 3.7 Model validation

Model predictions for A (water content), B (pH), and C (carbon to nitrogen ratio) were used to adjust the aged material. Because of composting *Streptomyces HN6* aged compost, a bacteriological fertilizer EC20 has been derived, containing 1.78 x 10^10^ CFU/g, with an effective bacterial content exceeding 1 x 10^9^ CFU/g, as per Chinese bio-fertilizer standards.

### 3.8 Application of bio-fertilizer

#### 3.8.1 Effect of bio-fertilizer on morphological indicators of cowpea

**Figure 4:**
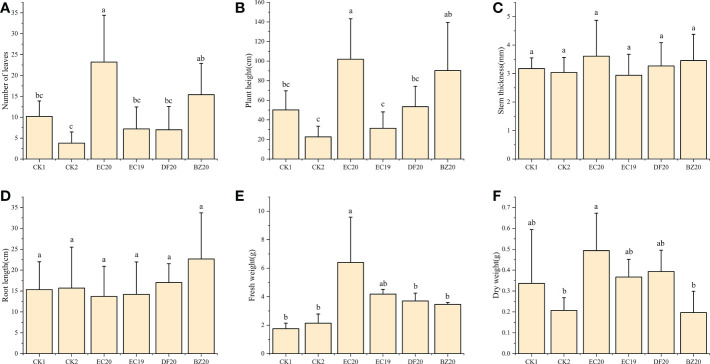
Effect of bio-fertilizer on phenotypic indicators of cowpea: **(A)** number of leaves; **(B)** plant height; **(C)** stem thickness; **(D)** root length; **(E)** fresh weight; **(F)** dry weight. Data represent the mean ± SD of at least three independent experiments, and different lowercase letters indicate that differences between different treatment groups reached significance at the P<0.05 level.

A response surface optimization study was carried out to determine EC19, BZ20, DF20, and EC20’s effect on cowpea plant growth on the 30^th^ day of inoculation with the cowpea wilt pathogen (**Figure 4**). The effect of EC19 on leaf counts was not significant, lower than EC20, and similar to controls without bacterial fertilizers CK1 (**Figure 4A**). A fungal fertilizer, EC20, had the greatest effect on plant height, followed by BZ20. The effects of the box-fermented fungal fertilizer, EC19, had little effect and was comparable with that of CK2, which was not fertilized and inoculated with cowpea blight (**Figure 4B**). A comparison of DF20 without *Streptomyces HN6* and CK1 without nutrients showed a similar result. This study finding suggests that *Streptomyces HN6* has a pro-growth effect on plant height (**Figure 4B**). Stem thickness and root length did not differ significantly between fertilizer treatments (**Figures 4C, D**). In comparison with CK1 and CK2, EC20 had the greatest impact on fresh weight, followed by EC19, which was also significant (**Figure 4E**). In EC20 treatment, the dry weight was significantly higher than in non-fertilized treatments, while in BZ20 treatment, it was lower but not significantly different from non-fertilized treatments (**Figure 4F**). The bacterial fertilizer EC20 significantly increased the number of leaves on the plants, the height of the plants, and the fresh weight of the plants throughout all the measurements above.

**Figure 5:**
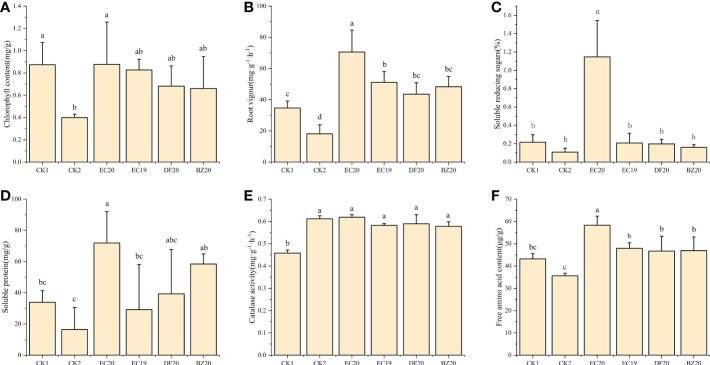
Effect of biofertilizer on physiological and biochemical indicators of cowpea: **(A)** chlorophyll content; **(B)** root vigor; **(C)** soluble reducing sugars; **(D)** soluble protein; **(E)** catalase activity; **(F)** free amino acid content. Data represent the mean ± SD of at least three independent experiments, and different lowercase letters indicate that differences between different treatment groups reached significance at the P<0.05 level.

Plants grow healthy when they have a high chlorophyll content in their leaves. The chlorophyll synthesis process in cowpea leaves will be disrupted or even destroyed when the disease affects photosynthesis efficiency, affecting the plant’s growth and development and decreasing its resistance to systemic diseases. Approximately 30 days after inoculation, cowpea leaves were collected from each treatment, and their chlorophyll content was measured. As a result of an EC20 treatment, the chlorophyll content was highest, followed by the sterile water control (CK1) with 0.876 mg·g^-1^ and 0.874 mg·g^-1^. In contrast, the control (CK2) treatment inoculated with cowpea blight had the lowest chlorophyll content at 0.40 mg·g^-1^ (**Figure 5A**).

Based on the root vigor index measured using the TTC method, the fungal fertilizer EC20 had the greatest impact on cowpea root vigor, while the other fungal fertilizers EC19, DF20, and BZ20 also contributed to plant root vigor, but did not differ significantly from CK1 (**Figure 5B**). It is also worth mentioning that EC20 significantly increased the soluble reducing sugar content of the plants compared to the other treatments, particularly compared to the pathogen control (CK2) by 958.5% (**Figure 5C**). Compared to the two controls, EC20 significantly increased the soluble protein content of plants, increasing it by 339.3% compared to the pathogenic control (CK2), and remained higher than the other two groups of natural compost substrates (DF20 and BZ20). However, there was no significant difference (**Figure 5D**). Each bacterial fertilizer compared with the control (CK1) that was not inoculated with pathogenic bacteria, did not significantly affect plant peroxidase. Catalase activity was increased because the pathogenic bacteria stimulated the cowpea seedlings, resulting in a stress response (**Figure 5E**). There was a significant increase in amino acid nitrogen content of the plants following treatment EC20 compared to the other treatments. In contrast, pathogen control (CK2) significantly decreased compared to the other treated groups. In EC20-treated plants, 63.8% of free amino acids were increased compared to pathogen-controlled plants (**Figure 5F**).

#### 3.8.2 Effect of bio-fertilizer on several key enzymes in the soil

**Figure 6:**
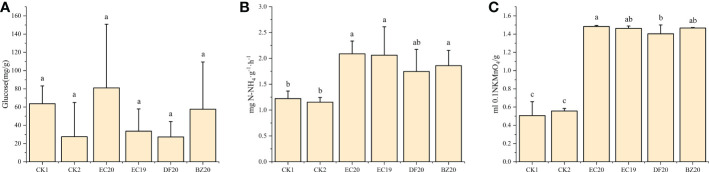
Effect of bio-fertilizer on soil enzyme activity: **(A)** soil transforming enzymes; **(B)** urease; **(C)** peroxidase. Data represent the mean ± SD of at least three independent experiments, and different lowercase letters indicate that differences between different treatment groups reached significance at the P<0.05 level.

The activities of three well-known soil enzymes, sucrase, urease, and catalase were evaluated. These enzymes are recognized as important indicators of soil fertility. A soil sample was taken from each treatment after 30 days of transplanting for analysis purposes. There was an increase in the EC20 soil conversion enzyme activity, but this difference was not significant compared to the other treatments. Bio-fertilizer treatments significantly differed from both controls regarding urease and catalase activities. An 81.7% increase in urease activity and a 164.3% increase in catalase activity were observed in the EC20 treatment compared to the cowpea blight group (**Figure 6**). The possible reason for this increase might be the correlation between the amount of substrate available for conversion by the microorganisms in the soil and the amount of urease activity in the substrate.

#### 3.8.3 Effectiveness of bio-fertilizer on cowpea blight prevention

**Figure 7:**
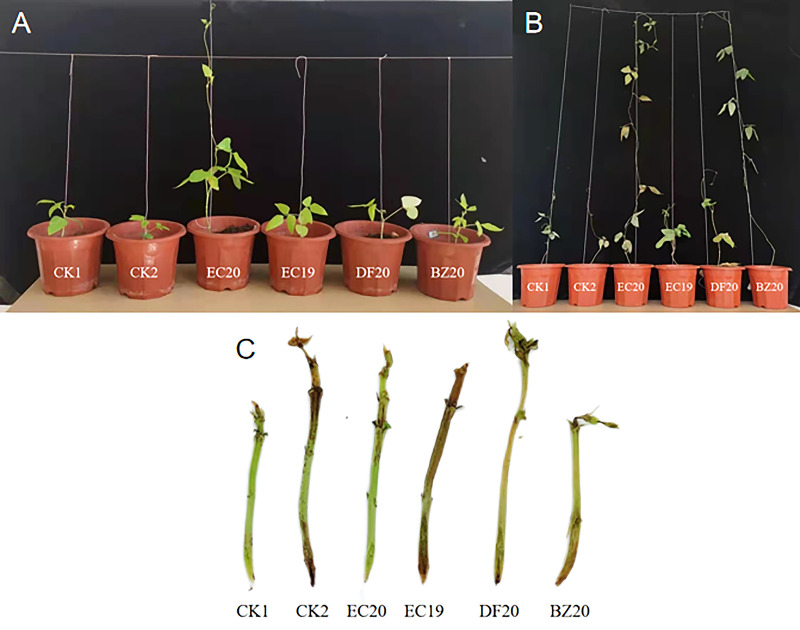
Effect of biofertilizer against cowpea blight on different plant stages and stem length. **(A)** Seedling stage **(B)** Adult stage, and **(C)** Stem length.

The obtained bacterial fertilizer was applied to the control of potted cowpea fusarium wilt. According to the results (**Table S4**, **Figure 7**), EC20 had the lowest disease incidence following the optimization of the response surface, and its disease index averaged 16.7, which was significantly lower than the other treatments. On cowpea, the bacterial fertilizer has a controlling effect of 78.45%; on the other hand, the natural fermentation fertilizer BZ20 has a wilting controlling effect of 59.09%. Also, the frame fermented bacterial fertilizer EC19 has a favorable control effect on cowpea wilting, with a control effect of above 50%; the uninoculated *Streptomyces HN6* bacterial fertilizer DF20 had a marginally substandard control effect, as it was only 20.38%. In this study, the bacterial fertilizer EC20 obtained by the natural composting method was found to be most effective in controlling cowpea fusarium wilt.

## 4 Discussion

A frame composting method, also known as vat composting, allows piles of compost to ferment in long vat composting frames, which are easy to turn and manipulate (Chang et al., 2021). Natural composting is the preparation of materials combined with site conditions piled into a certain shape fermentation composting way (De Corato, 2020). The advantage of this composting method is that it has low requirements for composting space and can be implemented according to local conditions (Bezabeh et al., 2021). Although these two composting methods are commonly used, there are few reports comparing the two methods in previous studies (Lau et al., 2009; Han et al., 2019). It is well known that its high temperature can kill pathogenic bacteria, weed seeds and other harmful substances, and accelerate physiological and biochemical reactions due to the exothermic nature of aerobic composting (Sahlström, 2003). Composting temperature is usually used as a progress index (Pezzolla et al., 2021). Generally, the composting temperature is higher than 55°C for 3 days to meet the composting requirements (Xu et al., 2019). In addition, electrical conductivity is related to small molecular soluble substances, which reflects the degree of mineralization of organic matter during fermentation (Cambardella et al., 2003). The ash content change reflects composting microorganisms’ decomposition and mineralization of compost substrate (Wang et al., 2015). Therefore, researchers often use composting temperature, conductivity and ash content as indicators to judge the composting effect. This study compared the effects of frame composting and natural composting on the final composting effect. The results showed that natural composting could produce higher pile temperature, and the duration of high temperatures was longer, the conductivity of the substrate changed more, and the ash produced during composting was also more than that of frame composting. Thus, natural composting is easier to accept by the public in the production, and the composting effect is more obvious. In this study, the application of the obtained bacterial fertilizer in cowpea planting was also studied. The results showed that the base material obtained by natural composting was more suitable for bacterial fertilizer. This result confirms the value of natural composting.

As an excellent microbial fertilizer inoculant, microorganisms have broad application space. Different microorganisms play an important role in different stages of composting (Nozhevnikova et al., 2019). Most actinomycetes are mesophilic bacteria, which play an important role in the aging period of compost (Soni and Devi, 2022). During this period, actinomycetes can not only accelerate the degradation of cellulose, hemicellulose and complex macromolecules but also change the structure of bacterial communities (Federici et al., 2011; Agnolucci et al., 2013). This was noted in this study when inoculating *Streptomyces HN6*. However, compared with other microbial inoculants, actinomycetes also has its own weaknesses; because of its slow growth rate. Most of the exogenously inoculated actinomycetes will lose their competitive advantage due to the insufficient number of bacteria, resulting in the phenomenon of colonization (Nakasaki et al., 1985). Previous studies have shown that the response surface method can be used to predict the appropriate growth conditions for the colonization of the strain (Miao et al., 2020). This study found that the response surface optimization of bacterial fertilizer EC20 has the ideal effect on disease prevention and growth promotion of potted cow. Further, several bacterial fertilizers without response surface optimization have no obvious effect on plant growth promotion. For example, the effect of EC19 on leaf number and plant height was not significantly different from that of CK1 without any bacterial fertilizer, while the dry weight of plants treated with BZ20 was even lower than that without bacterial fertilizer. It can be seen that the successful preparation of bacterial fertilizer is related to the inoculation conditions, and the response surface method can help us solve this problem.

A previous study on *Streptomyces HN6* wettable powder (Luo et al., 2018), demonstrated a certain control effect on soil-borne diseases of plants; however, some negative additives were inevitably added in the preparation process. The bacterial manure we obtained in this study was developed with straw as compost base. It not only has good disease prevention effect, but also has fertilizer effect, which can promote the growth of cowpea. The application of bacterial manure not only contributes to the green prevention and control of cowpea wilt, but also provides a new way for straw waste utilization. However, the obtained bacterial fertilizer has not been widely applied to more crops in our current study. Therefore further studies are required to investigate its use in a wide range of crops.

## 5 Conclusion

*Streptomyces* sp. *HN6*, a biocontrol strain that promotes growth and disease prevention, was used as an additive strain in this study to compare the composting effects of frame composting and natural composting. Results demonstrated that natural composting is more suitable for promotion in the field, a fertilizer obtained after response surface optimization (EC20) was the most effective against cowpea wilt. This bio-fertilizer significantly promotes plant growth, increased number of leaves, and plant height and fresh weight of the plant. Additionally, this bio-fertilizer significantly increased soil-transforming enzyme activities, particularly urease and catalase, which were significantly higher than in control. The results of this study indicate that *Streptomyces HN6* bio-fertilizer could potentially replace conventional fertilizer in many situations.

## Data availability statement

The original contributions presented in the study are included in the article/**Supplementary Material**. Further inquiries can be directed to the corresponding authors.

## Author contributions

TW and SA: conceptualization, methodology, software, investigation, formal analysis, and writing—original draft. LY, XY, YZ, and SZ: software, investigation, formal analysis, and writing—revised draft. LW and YL: conceptualization, funding acquisition, resources, supervision, and writing—review and editing. All authors contributed to the article and approved the submitted version.
