# Supplementary material for: Preparation, biocontrol activity and growth promotion of biofertilizer containing Streptomyces aureoverticillatus HN6
Source: Front Plant Sci. 2022 Dec 15;13:1090689. doi: 10.3389/fpls.2022.1090689 (PMC9798099; doi:10.3389/fpls.2022.1090689)
Supplement: Supplementary file 1 [file DataSheet_1.docx]

**Running Title: Preparation, Biocontrol Activity and Growth Promotion of Biofertilizer**

**Preparation, Biocontrol Activity and Growth Promotion of Biofertilizer containing *Streptomyces aureoverticillatus* *HN6***

**Tianhao Wang^†^, Shakil Ahmad^†^, Lin Yang, Xiangnan Yan, Yunfei Zhang, Shujing Zhang, Lanying Wang*, Yanping Luo***

School of Plant Protection, Hainan University, Haikou, Hainan, 570228, China

***Corresponding Authors:**

Yanping Luo

[yanpluo2012@hainanu.edu.cn](mailto:yanpluo2012@hainanu.edu.cn)

Lanying Wang

[daivemuwly@126.com](mailto:daivemuwly@126.com)

**^† These authors have contributed equally to this work and share first authorship.^**

**Table S1. Response surface experimental factor level design table**

| Level Factor | Factors | | |
| --- | --- | --- | --- |
|  | Water content（%） | pH | C/N |
| -1 | 40 | 6 | 20 |
| 0 | 50 | 7 | 25 |
| 1 | 60 | 8 | 30 |

**Table S2. Experimental results of response surface analysis**

| **Number** | **A**  **Water content (%)** | **B**  **pH** | **C**  **C/N** | **Y**  **Bacterial content （10^9^cfu·g^-1^）** |
| --- | --- | --- | --- | --- |
| 1 | 40 | 8 | 25 | 0.75 |
| 2 | 50 | 6 | 30 | 0.50 |
| 3 | 50 | 8 | 30 | 1.00 |
| 4 | 40 | 7 | 30 | 0 |
| 5 | 60 | 6 | 25 | 0.25 |
| 6 | 60 | 7 | 20 | 0.25 |
| 7 | 50 | 7 | 25 | 1.50 |
| 8 | 60 | 7 | 30 | 0.25 |
| 9 | 50 | 6 | 20 | 0.50 |
| 10 | 50 | 8 | 20 | 0.50 |
| 11 | 50 | 7 | 25 | 1.75 |
| 12 | 40 | 6 | 25 | 0 |
| 13 | 50 | 7 | 25 | 1.00 |
| 14 | 50 | 7 | 25 | 1.75 |
| 15 | 50 | 7 | 25 | 1.25 |
| 16 | 40 | 7 | 20 | 0.25 |
| 17 | 60 | 8 | 25 | 0.50 |

Regression analysis of the test data using the Design-Expert 8.0 software program yielded the following regression equation for the amount of bacteria contained in secondary fermentation 0 Streptomyces HN6:

Y= -45.8 + 8.1A + 46.5B + 7.8C - 0.13AB + 0.013BC - 0.25BC - 0.08 A2 - 0.32 B2 - 0.02 C2

Where A indicate water content, B indicate pH and C indicate carbon to nitrogen ratio, with a correlation coefficient of R=0.9480.

**Analysis of variance**

The results of ANOVA and goodness-of-fit test on the regression equation are shown in Table S3. ≈ C (carbon to nitrogen ratio).

**Table S3. Analysis of variance for secondary response surface regression models**

| **Source of variance** | **Sum of squares** | **DF** | **Mean Square** | **F** | **Prob>F** | **Significance** |
| --- | --- | --- | --- | --- | --- | --- |
| Model | 4.75×10^18^ | 9 | 5.27×10^17^ | 6.91 | 0.0092 | ** |
| A - Water content (%) | 7.81×10^15^ | 1 | 7.81×10^15^ | 0.1 | 0.7584 |  |
| B-pH | 2.81×10^17^ | 1 | 2.81×10^17^ | 3.68 | 0.0964 | * |
| C-C/N | 7.81×10^15^ | 1 | 7.81×10^15^ | 0.1 | 0.7584 |  |
| AB | 6.25×10^16^ | 1 | 6.25×10^16^ | 0.82 | 0.3956 |  |
| AC | 1.56×10^16^ | 1 | 1.56×10^16^ | 0.2 | 0.6647 |  |
| BC | 6.25×10^16^ | 1 | 6.25×10^16^ | 0.82 | 0.3956 |  |
| A^2 | 2.41×10^18^ | 1 | 2.41×10^18^ | 31.54 | 0.0008 | ** |
| B^2 | 4.28×10^17^ | 1 | 4.28×10^17^ | 5.6 | 0.0498 | * |
| C^2 | 1.08×10^18^ | 1 | 1.08×10^18^ | 14.14 | 0.0071 | ** |
| Residuals | 5.34×10^17^ | 7 | 7.63×10^16^ |  |  |  |
| Loss of proposed items | 1.09×10^17^ | 3 | 3.65×10^16^ | 0.34 | 0.7969 |  |

Note:*Significant at p<0.05,**Significant at p<0.01

**Table S4. Efficacy of different compost products against cowpea**

| **Treatment name** | **Disease index** | **Effectiveness (%)** |
| --- | --- | --- |
| CK1 | - | - |
| CK2 | 77.5±7.5 a | - |
| EC20 | 16.7±6.3 d | 78.45 |
| EC19 | 37.5±2.5 c | 51.61 |
| DF20 | 61.7±5.2 b | 20.38 |
| BZ20 | 31.7±3.8 c | 59.09 |
